# FRIEND Engine Framework: a real time neurofeedback client-server system for neuroimaging studies

**DOI:** 10.3389/fnbeh.2015.00003

**Published:** 2015-01-30

**Authors:** Rodrigo Basilio, Griselda J. Garrido, João R. Sato, Sebastian Hoefle, Bruno R. P. Melo, Fabricio A. Pamplona, Roland Zahn, Jorge Moll

**Affiliations:** ^1^Cognitive and Behavioral Neuroscience Unit and Neuroinformatics Workgroup, D'Or Institute for Research and EducationRio de Janeiro, Brazil; ^2^Center of Mathematics, Computation and Cognition, Universidade Federal do ABCSanto André, Brazil; ^3^Department of Psychological Medicine, Institute of Psychiatry, King's CollegeLondon, UK

**Keywords:** brain computer interface (BCI), real-time fMRI, FSL, neurofeedback, EEG

## Abstract

In this methods article, we present a new implementation of a recently reported FSL-integrated neurofeedback tool, the standalone version of “Functional Real-time Interactive Endogenous Neuromodulation and Decoding” (FRIEND). We will refer to this new implementation as the FRIEND Engine Framework. The framework comprises a client-server cross-platform solution for real time fMRI and fMRI/EEG neurofeedback studies, enabling flexible customization or integration of graphical interfaces, devices, and data processing. This implementation allows a fast setup of novel plug-ins and frontends, which can be shared with the user community at large. The FRIEND Engine Framework is freely distributed for non-commercial, research purposes.

## Introduction

There is a growing push toward the use of real-time fMRI (rt-fMRI) neurofeedback in experimental and clinical investigation, with solid prospects for therapeutic applications (Sulzer et al., 2013; Stoeckel et al., 2014) through the development of brain computer interface (BCI) software integrated with commonly available devices such as fMRI scanners and EEG devices. Successful scientific exploration and application of rt-fMRI neurofeedback will be facilitated by the existence of freely available, user-friendly, and flexible software implementations. To this end, a few research groups have recently contributed with the development of computational tools, each with their own strengths and limitations (LaConte et al., 2007; Zotev et al., 2011; Sorger et al., 2012; Rana et al., 2013; Sato et al., 2013).

Nevertheless, because of the computational and technical complexities that are intrinsic to the emerging field of rt-fMRI neurofeedback and the diversity of approaches, there are still few available options for investigators in the area. More importantly, a number of features that could facilitate and encourage advanced users and developers to build on currently available platforms are still lacking. Here, we introduce a new framework that unleashes users to create frontends and customize pipelines for their own studies using the programming language and platform they are most familiar with, flexibly connecting them to a core processing engine. To attain this goal, we have revamped our recently reported rt-fMRI neurofeedback standalone software, “Functional Real-time Interactive Endogenous Modulation and Decoding system” (FRIEND) (Sato et al., 2013), as described below.

## FRIEND engine framework

**Figure 1:**
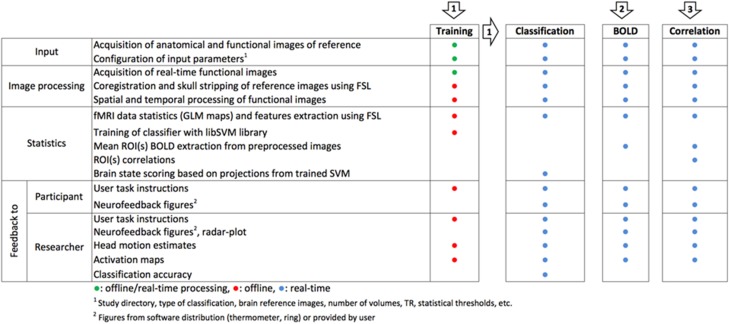
**Flowchart of three FRIEND processing neurofeedback pipelines**. (1) Support Vector Machine-based neurofeedback, using projected values onto a discriminative hyperplane; (2) BOLD level real-time display from a set of pre-specified ROIs; (3) Real-time functional connectivity neurofeedback based on the correlation between the signals from different ROIs (Sato et al., 2013).

The original FRIEND neurofeedback tool comprises three processing pipelines (Figure 1): (1) the brain decoding-based feedback using support-vector machines (SVM); (2) the single region-of-interest (ROI) based blood-oxygen level dependent (BOLD) feedback; and (3) functional connectivity feedback based on a sliding window of correlations between ROIs. These pipelines were developed and implemented across time, on the basis of research needs in our lab, including the techniques needed to support these pipelines. As such, the first version of FRIEND included only the SVM pipeline for brain decoding-based neurofeedback for emotion modulation, using the libSVM library (Chang and Lin, 2011) and was recently employed in a study of emotional enhancement (Moll et al., 2014). For the purpose of a new research line on motor control physiology and rehabilitation, an ROI activation pipeline for FRIEND was implemented, allowing the flexible creation of anatomically or functionally defined ROIs. Anatomical ROIs are created by selecting predefined areas from a variety of Montreal Neurological Institute (MNI) templates, which are automatically transformed into the subject space. Functionally-defined ROIs can also be created after running an fMRI functional localizer, and then selecting and thresholding a functional cluster for subsequent neurofeedback runs. The third pipeline, focusing on sliding window-based correlations between two brain regions as a guide for neurofeedback information, was developed to enable an ongoing clinical proof-of-concept study on remitted major depression, based on the findings of a recent study (Green et al., 2012).

The provision of the ideal feedback to the participant is crucial for the success of any neurofeedback study. In this vein, interoperability with other applications such as well-established stimulus presentation tools, including virtual reality ones, and response devices with the neurofeedback interface would be highly desirable (Renaud et al., 2011; Weiskopf, 2012). In the original implementation of FRIEND (Sato et al., 2013), a few standard strategies were implemented, based on a simple visual feedback interface (e.g., rings changing in shape or a thermometer). In that original implementation, the visual feedback could be modified by simply replacing the bitmap figures. However, this initial approach neither allows for a more sophisticated control of stimuli as specialized stimulus presentation softwares do (e.g., http://www.neurobs.com/), nor does it offer the possibility of immersive game-like experience, which could be more engaging for participants of neurofeedback studies.

In order to allow users to use their own stimulus feedback strategies and to develop additional processing strategies (e.g., Matlab® pipelines), we have broken the standalone version of FRIEND into smaller parts, encapsulating the complex calculations involving rt-fMRI neurofeedback processing in one unit and the graphic user interface in another. We expect that this approach will enable researchers with average to advanced programming skills to efficiently implement customized graphical interfaces and data processing functionalities, which can be shared with other users via NITRC (http://www.nitrc.org/projects/friend) and GitHub (https://github.com/InstitutoDOr/FriendENGINE). Toward this aim, FRIEND was restructured based on the well-established object oriented programming paradigm (Stroustrup, 1988), facilitating modification and implementation of new functionalities.

**Figure 2:**
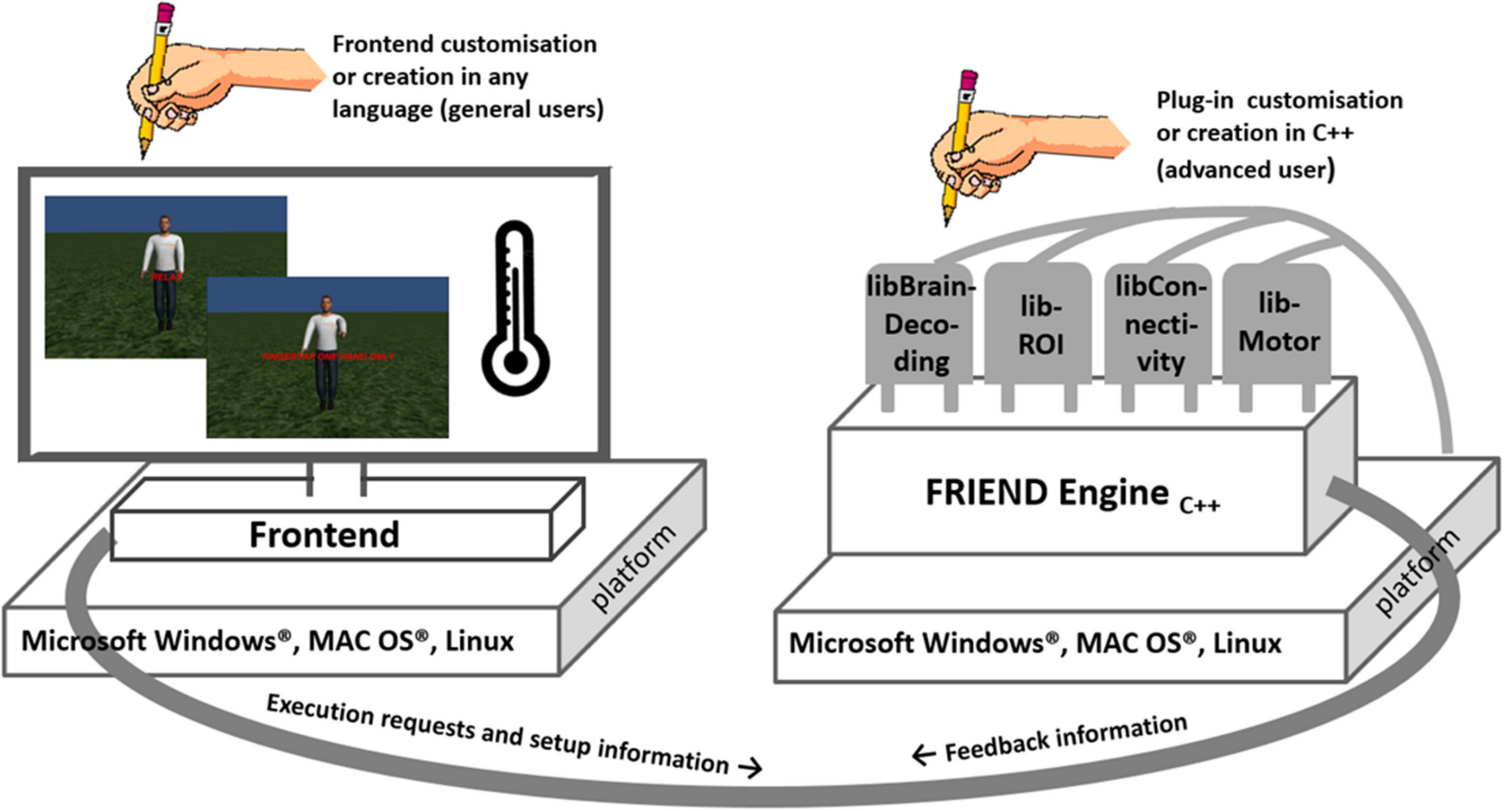
**The FRIEND Engine framework comprises three major components**. The FRIEND Engine core and plug-ins, written in C++ and operating on the same platform, and the frontend that can be written in any language with sockets support. The TCP/IP communication protocol allows the frontend to be executed on a different computer platform from the FRIEND Engine. Users may customize or write their own frontends. Advanced users can also write their own plug-ins and processing pipelines.

In FRIEND Engine, basic processes that are common to any neurofeedback pipeline such as anatomical segmentation, motion correction of functional images and gaussian smoothing (Figure 1)—largely based on FSL code (http://fsl.fmrib.ox.ac.uk/fsl/fslwiki/)—are encapsulated in a single unit, called FRIEND Engine (Figure 2), the first part of the FRIEND Engine framework. The engine determines the main processing pipeline and defines time points in this pipeline to execute functionalities not implemented in the engine. These functionalities are geared to attain the goal of the study (e.g., classification of brain states, ROI percent signal change and correlation between two ROIs in the previously presented pipelines) and coded separately in independent units, called plug-ins (Figure 2) here, the second component of the framework. By definition, a plug-in is an extension of the engine and must therefore be implemented in the same language and be executed on the same platform for compatibility. As such, the engine provides the basic workings whereas the plug-ins implement the specific functionalities necessary for estimation of feedback parameters on the basis of data analysis. The engine does not know and does not need to know how a specific plug-in handles the information. The plug-in library just needs to expose the necessary functions for the engine to work properly. The coordinator of the engine and the neurofeedback processing is the frontend (Figure 2), the third part of the FRIEND Engine framework. As the name intuitively implies, it is the graphical interface for the neurofeedback loop, essentially the *interface* of a BCI. It provides feedback information (e.g., BOLD activation level) to the participant, relevant information to the operator (e.g., motion correction parameters), and handles the necessary steps to synchronize the presentation of the feedback with the scanner acquisition. Ideally, the construction of the frontend should not demand learning new and complex technical skills. The optimal solution is allowing users to customize their applications using the programming language and platform of their choice. A TCP/IP network communication protocol is therefore defined to bridge the frontend and the engine. In this configuration, the engine listens to a port for incoming messages (requests) from the frontend, allowing the engine and frontend to operate on different platforms.

The first step of a neurofeedback study in the FRIEND Engine framework is the same as for the standalone version of FRIEND: the configuration of the specific parameters for the study, such as the input directory and the number of volumes in the acquisition run. This configuration should be provided by the frontend application, which gets the input from the operator of the study and passes it to FRIEND Engine. By default, FRIEND Engine reads the study_params.txt configuration file located in the same directory of the engine's executable file. The study_params.txt file is exactly the same as for the standalone version of FRIEND. The frontend can pass a whole configuration file by the TCP/IP network communication protocol to the engine by way of the command “READCONFIG,” as explained in the following section. The next important and vital command the frontend must pass on, is the plug-in configuration, which comprises the plug-in library filename and the name of the functions that the engine should call at predefined time points, like the name of the function that calculates the feedback information for a volume. These two messages prepare the engine to properly handle the experiment. The next message the frontend should send is “PREPROC,” which executes the same steps executed in the standalone version of FRIEND after the first configuration window.

Next, the frontend needs to send a message indicating that the engine should start processing the acquisition run. There are four options, “PIPELINE,” “NBPIPELINE,” “FEEDBACK,” “NBFEEDBACK” (Table 1), explained in the following section. This is equivalent to clicking the “TRAIN” or “FEEDBACK” button in the standalone version of FRIEND. At specific points of the processing, such as the calculation of the feedback, the engine executes the proper plug-in function, previously assigned to the plug-in configuration phase. There is no direct communication between the frontend and the plug-in components.

**Table 1:** **List of commands expected by FRIEND Engine during the TCP/IP communication protocol with the frontend**.

**Command**	**Action**
PREPROC/NBPREPROC	Performs the initial preprocessing (co-registration, skull stripping, MNI registration, etc.) steps of FRIEND
PIPELINE/NBPIPELINE/NBFEEDBACK	Starts the processing of each volume in the acquisition run as soon as it becomes available in the input directory. The difference between the PIPELINE/NBPIPELINE and NBFEEDBACK commands, is that NBFEEDBACK automatically calculates the feedback values and stores them in a session workspace after the processing of each volume
GLM/NBGLM	Performs the General Linear Model (fsl_glm) calculation using the FSL toolbox
FEATURESELECTION/NBFEATURESELECTION	Performs feature selection (see Sato et al., 2013), i.e., identifies the most representative subset of voxels for the calculation of the feedback value
PLUG-IN	Defines the library and the associated plug-in functions to be used in further calls
TRAIN/NBTRAIN	Calls the train function plug-in
TEST	Calls the plug-in feedback function
NEWSESSION	Creates a new session (workspace) in the engine memory
ENDSESSION	Indicates that the engine can terminate an opened session and closes the related thread
SESSION/GRAPHPARS	Queries for the motion parameters of a given volume
SESSION/TEST	Returns the feedback information of a volume, previously stored in the session workspace
SESSION/PREPROC, SESSION/FEEDBACK	Queries if a command, e.g. PREPROC or FEEDBACK has ended
READCONFIG	Sends an entire configuration file associated with the frontend neurofeedback study to set the parameters of the experiment

**Figure 3:**
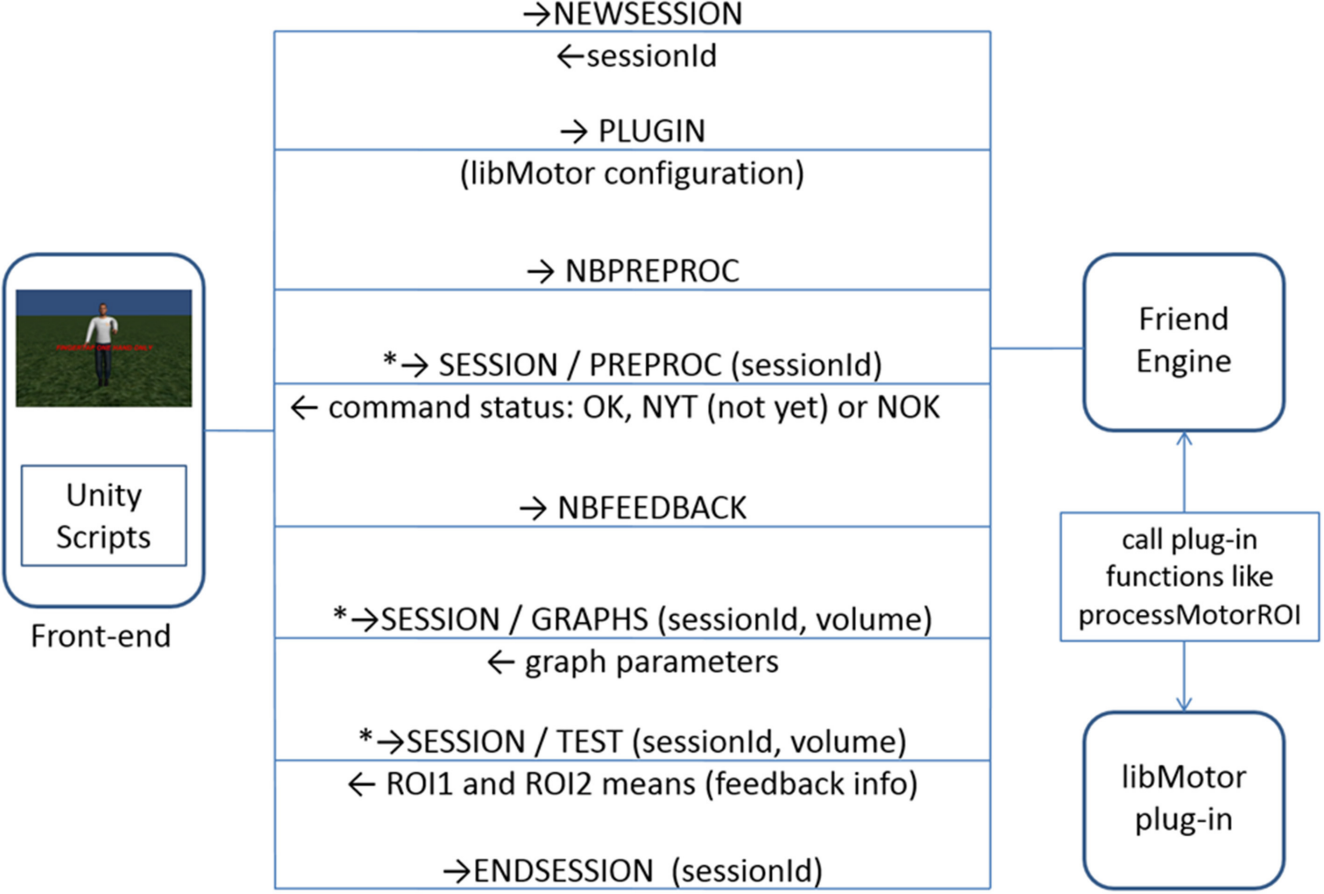
**TCP/IP network communication protocol between the frontend and the FRIEND Engine (avatar finger tapping virtual scenario)**. The steps marked with asterisk (^*^) are called as many times as needed. The first message sent is “NEWSESSION,” which indicates that the engine should create a new session to work with the frontend. That message is only needed in asynchronous communications as explained in Section TCP/IP Communication Protocol.

During the acquisition run, the frontend sends “TEST” messages (Table 1), querying for neurofeedback information for each volume of the acquisition scan. The engine executes the configured feedback function of the plug-in to get the feedback information and returns it to the frontend. The frontend must interpret this value and properly display that information to the participant of the experiment. Figure 3 depicts this message exchange between the frontend and the engine in the avatar finger tapping virtual scenario. It includes a new command, “NEWSESSION” (Table 1), which indicates that the engine should create a new session to work with the frontend. That message is only needed in asynchronous communications as explained in Section TCP/IP Communication Protocol. To illustrate this, excerpts of programming code from a Matlab® frontend and the libROI plug-in are provided in the Supplementary Material. FRIEND Engine expects the volume files in exactly the same way as standalone FRIEND does. This implies that the list of computers that can run the engine is restricted to the list of computers that can receive the volume files from the fMRI scanner (or EEG device) in real-time acquisition.

FRIEND Engine runs on Microsoft Windows® (XP or later), Apple Macintosh (OSX 10.8 and above) and Linux (Debian, CentOS 6.4). A mid/high end workstation is required (e.g., PC: quad-core i7, 8 GB RAM or higher, Macintosh: quad-core Intel Core i5, 8 GB RAM or higher). In the original standalone version of FRIEND, FSL (Jenkinson et al., 2012) toolbox commands were encapsulated in a dynamic link Microsoft Windows® library. In FRIEND Engine, this interrelationship was changed: for non-Microsoft Windows® systems, FSL toolbox installation is a pre-requisite and FSL executables are called using system calls to the operational system. This simplifies FSL upgrades, as they can be executed independently from the engine code. In Microsoft Windows® FRIEND Engine version, our modified source code of the FSL toolbox functionality is embedded within the executable of FRIEND Engine. This embedding process transforms each needed FSL command in a function statically linked to the software. For this reason, no additional installation of the FSL software is necessary.

### TCP/IP communication protocol

Table 1 lists the commands expected by the engine in the TCP/IP communication protocol. There are two types of connections: synchronous (blocked) and asynchronous (non-blocked [NB]) connections. In synchronous connections, the frontend needs to wait until command completion to receive the acknowledge response from the engine. Asynchronous connections need to be established when the frontend is not supposed to freeze the execution to wait for the acknowledge response, as in virtual reality scenario frontends. This situation happens when a time-consuming command needs to be executed, like “PREPROC” or “TRAIN.” In this case, regular queries for command termination need to be issued until the expected response is obtained. To appropriately handle asynchronous connections, a multithread approach with at least two threads is adamant: one, the main thread, that performs all the raw real time processing; and the other, the response thread, which responds to queries of various types of information related to the main thread processing. The two threads can respond properly to the frontend by a shared access to the same session. To make these two threads interoperate, the notion of session is introduced. A session is an independent location in the memory of the computer running the engine, capable of storing all the information that is required to be sent back to the frontend, such as neurofeedback information and motion-corrected volume parameters. It is a role of the frontend to present this information to the participant in a user-friendly manner. The Matlab® frontend provided with the software distribution has some built-in capabilities to display feedback and motion parameters.

### Plug-in library

A plug-in file is a dynamic library file (a.so file on the Linux system, a.dylib file on the Mac OSX system and a.dll file in Microsoft Windows®) that implements specific functions called internally by FRIEND Engine at specific times during the pipeline. This is a major advantage of the FRIEND Engine, because, when in need of additional features, users can focus on writing just the necessary functionality for their specific research needs. This allows customization of the neurofeedback tool, using encapsulated codes that run additional functionalities from external libraries, leaving the engine code intact. This characteristic favors usability and code maintenance, because errors in a plug-in library are also encapsulated in that library and do not affect other plug-ins. This framework makes it easier to setup pilot experiments and to explore new hypotheses.

#### Plug-in functions and parameters

The plug-in library must implement all the computational processes required to calculate the feedback responses. A small subset of variables needs to be defined to be used as parameters of the plug-in functions (Table 2).

**Table 2:** **List of parameters used within plug-in functions**.

**Parameter**	**Definition**
studyParams	Object that stores information from the study_params.txt file, which encloses all the information used by the processing pipeline to correctly identify the files, directories and expected number of data volumes
userData	Pointer referencing an area of memory created by the engine to be used by the plug-in for temporary calculations. The reason behind this approach is that subsequent calls to the same plug-in from different frontends would overwrite internal data structures if they were created within the plug-in library
classNum	Number of the class, i.e., the condition attributed to the scan during classification
volumeIndex	Number of the current processed scan
volumeFileName	The filename of the volume in the file system
Projection	Feedback value calculated
Index	Number of current scan

The engine defines six functions (Table 3) that are called at predefined time points during the pipeline execution. Not all of those six functions must be implemented in a plug-in library, just the ones necessary to properly calculate the feedback information. We recommend advanced users to code those functions in C++ because that minimizes compatibility errors during the execution of the plug-in functions by the engine.

**Table 3:** **List of functions a plug-in can define**.

**Function**	**Definition**	**Parameters (Table 2)**
Train	Executed when the frontend issues the TRAIN command (Table 1). Normally, this function is used to analyze a complete data set (e.g. a training run). This function is generally more time consuming, and is therefore not a “real-time” operation. It is normally called at the end of the acquisition of a training run (e.g., for a multi voxel pattern analysis [MVPA] classification approach) or a functional localizer (e.g., for an ROI-based BOLD signal or functional connectivity study). This function builds a model that can be used on subsequent runs	studyParams userData
Test	Executed when the engine needs to calculate the feedback value for a given volume. There are two predefined values that the engine returns to the frontend: information about the condition of the processed image volume indicating, for example, a specific type of image volume classification in a MVPA study, and the feedback information value. The interpretation of these two variables is left to the frontend, which must implement how this information will be conveyed to the participant (e.g., a thermometer level, changes in a visual or auditory feedback)	studyParams userData classnum projection index
Initialization	Called after the engine reads information from the study configuration file (see Sato et al., 2013). All the memory data structure used by the plug-in must be initialized here. A pointer reference for this data structure has to be returned in the function argument. This reference will be used in further plug-in function calls issued by the FRIEND Engine	studyParams userData
Finalization	Called right before ending the processing of a session. All the memory data structures allocated in the Initialization function must be destroyed here	studyParams userData
Volume	Called before the processing of a volume	studyParams volumeIndex volumeFilename userData
PostProc	Called after the pre-processing of each volume	studyParams volumeIndex volumeFilename userData

### Available plug-ins

The FRIEND Engine distribution comes with four plug-ins: one for the SVM pipeline (libBrainDecoding), using the libSVM library (Chang and Lin, 2011); one for the ROI pipeline (libROI), used in the Matlab® and the first game frontend examples (presented in the following sections); one for the functional connectivity between two ROIs (libConnectivity) and one (libMotor) that extracts ROI information from two ROIs located in the motor cortex area (left and right), used in the avatar finger tapping virtual scenario.

#### libROI plug-in functions and feedback value

Table 4 lists functions implemented in the libROI plug-in. The feedback value calculated by the processROI function (Table 4) is given by the equation:

(1) $$\frac{{\bar{ROI}}_{curr\_vol}-\frac{1}{B}\sum_{k\;=\;1}^{B}{\bar{ROI}}_{k}}{\frac{1}{B}\sum_{k\;=\;1}^{B}{\bar{ROI}}_{k}}$$

where *ROI*_*curr_ vol*_ is the mean of the ROI on the current volume, *B* is the number of volumes in the previous baseline condition and ROI_*k*_ is the mean of the *k*th volume.

**Table 4:** **Functions implemented in the libROI plug-in**.

**Plug-in**	**LibROI**
initializeROIProcessing	Initialization function that creates the data structures necessary for processing ROIs. It also transforms the user-defined MNI ROI mask into subject space
processROI	Feedback function that calculates the percent signal change of a ROI in the current volume compared to the mean activation of a baseline block
finalizeROIProcessing	Finalization function that destroys all data structures created in the initialization function

A code snippet of this function can be found in the Supplementary Material. The feedback function within the libMotor plug-in is similar to the one on libROI, except for the fact that two feedback values are calculated, one for each existing ROI.

#### libBrainDecoding plug-in functions and feedback value

Table 5 lists functions implemented in the libBrainDecoding plug-in. In trainSVM function, the voxels of an fMRI scan are first organized (by concatenation) in an input vector *x*. In this training phase, the vector is labeled according to the corresponding experimental condition (LaConte, 2011; Sitaram et al., 2011). This initial data is used to train the classifier (currently, a two-class SVM classifier is implemented) to discriminate between the experimental conditions of interest. The output of this function is the trained SVM model (i.e., the hyperplane coefficients). The trained SVM is then used in the subsequent brain decoding sessions (testing sessions), during which participants engage in the same tasks and conditions of interest.

**Table 5:** **Functions implemented in the libBrainDecoding plug-in**.

**Plug-in**	**LibROI**
trainSVM	Function that uses the libSVM library to train the SVM model
testSVM	Function that calculates the feedback value of a scan based on its projected values on an SVM hyperplane
initSVM	Initialization function that creates the data structures required for SVM training and testing
finalSVM	Finalization function that deletes all temporary data structures created by initSVM

In testSVM function, the projected value of a new observation is used to define the neurofeedback information (Sato et al., 2008). The projected value of a new image volume on the SVM discriminating hyperplane is given by (*x^T^w* + *b*), where *w* is a vector containing the hyperplane coefficients and *b* is a constant. The boundary between conditions is represented by the value of zero. This value obtained by projecting the new observation in the SVM discriminating hyperplane is then used to choose the feedback figure to be shown to the participant. Further information can be found in Sato et al. (2013).

#### libConnectivity plug-in functions and feedback value

Table 6 lists functions implemented in the libConnectivity plug-in. The buildROIs function transforms a mask with two MNI ROIs into the subject space. A subset of voxels from a GLM analysis of the localizer run is then selected by applying the preceding masks and selecting a user-defined percentage of these voxels. These two voxel populations are employed as new ROIs for the sliding window correlation calculation.

**Table 6:** **Functions implemented in the libConnectivity plug-in**.

**Plug-in**	**LibROI**
buildROIs	Training function responsible for building a mask containing two ROIs used to calculate the associated correlations
calculateFeedback	Feedback function that calculates the sliding window correlations based on the two ROIs calculated in buildROIs function
initializeFunctionalConnectivity	Initialization function that creates the necessary data structures
finalizeFunctionalConnectivity	Finalization function that destroys all data structures created by initializeFunctionalConnectivity function

The calculateFeedback function calculates the Pearson correlation coefficient between two ROIs, where *ROI*1 and *ROI*2 are vectors containing the means of the ROIs on the last *L* scans:

(2) $$\begin{array}{l} \rho (ROI1,ROI2)=\frac{\sum_{i\;=\;1}^{L}\left(ROI1_{i}-\bar{ROI1}\right)(ROI2_{i}-\bar{ROI2})}{\sqrt{\sum_{i\;=\;1}^{L}{\left(ROI1_{i}-\bar{ROI1}\right)}^{2}}\sqrt{\sum_{i\;=\;1}^{L}{\left(ROI2_{i}-\bar{ROI2}\right)}^{2}}}\;\;\;\; \end{array}$$

Where *L* is the size of the sliding window, i.e., the last *L* scans acquired.

## Frontend examples

In all the “game” examples provided with the distribution, there is, in the engine directory, a pre-configured study_params.txt file that is read by default by the engine; all the volume files are already placed in the input directory referenced by the study_params.txt file. To read the volume files in a real-time online setup, users need to configure the arrival of images in the input directory in the same way as for the standalone version of FRIEND. Triggers from the scanner can be used to keep track of the time, e.g., the onset of a given experimental condition. It is the frontend that handles the syncing of the experimental paradigm with the scanner.

### Matlab® frontend

**Figure 4:**
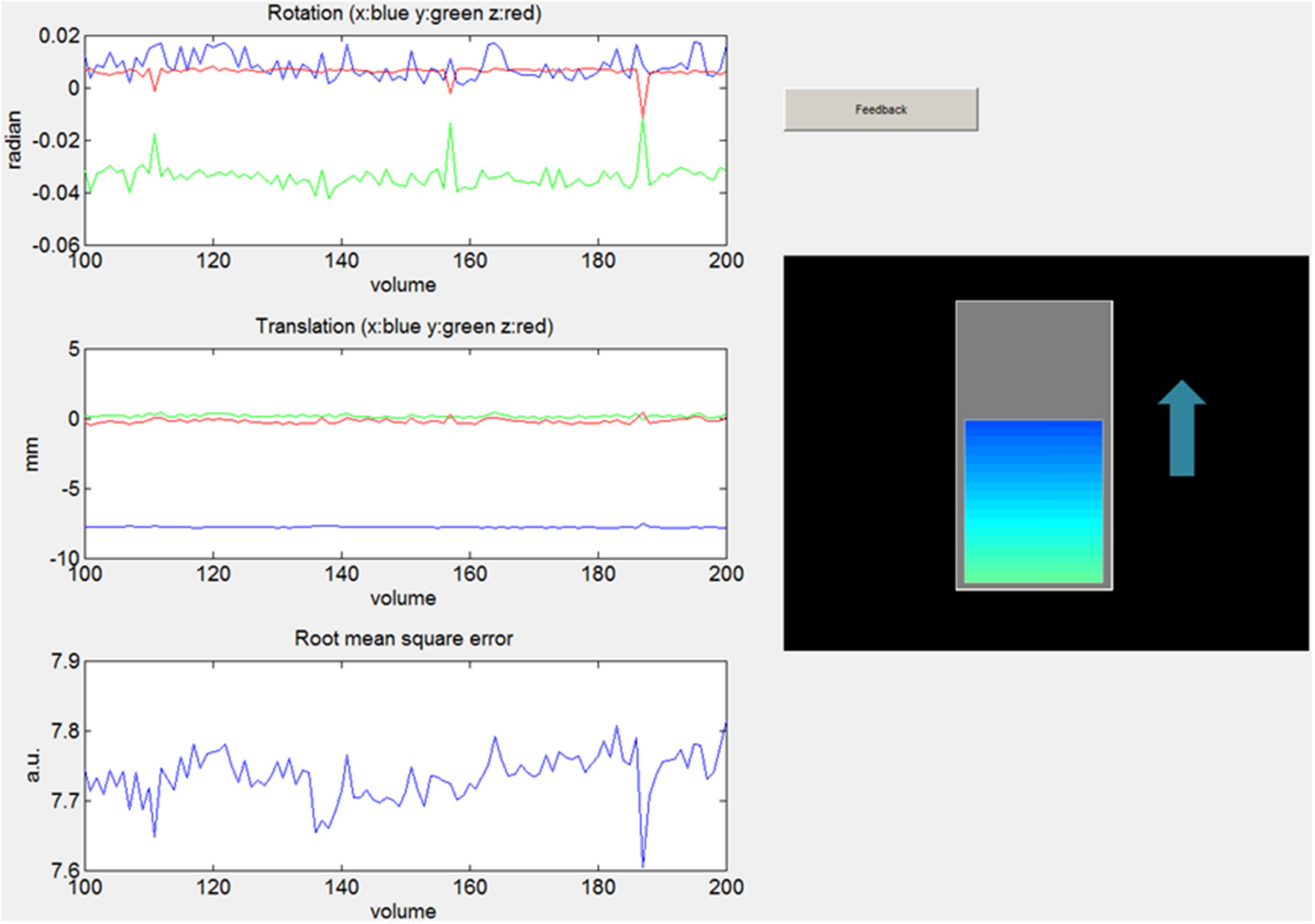
**Snapshot of a frontend designed with the Matlab® GUIDE tool, showing motion parameters (rotation, translation and root mean square error) and a thermometer indicating BOLD signal change as a feedback**.

Figure 4 is a screenshot of a frontend designed with the Matlab® GUIDE tool. Matlab® is a largely used language in the scientific community so it is important to provide a functional example of the Matlab® FRIEND Engine connection. GUIDE helps users to build graphical user interfaces for their applications in Matlab®.

This frontend application shows the activation of a ROI in the motor cortex during a finger-tapping task. It presents the feedback in the shape of a thermometer-like dynamic bar graph. This example uses the libROI plug-in. In this example and the first Unity example below, only one ROI located in the left motor cortex is used.

### Unity frontends

The frontends based on the Unity game engine (http://unity3d.com/) employ virtual reality scenarios. These scenarios are composed of objects, like rocks, trees and scripts. The scripts in Unity play an important role in how the virtual scenarios behave, such as the interactions between objects, and how, where and when avatars interact with the environment. This aspect is especially relevant for the interrelationship between Unity and FRIEND Engine. Scripts coordinate how the information returned by the engine will impact on the current state of the virtual scenario. The complexity of this coordination increases exponentially with the number of objects and avatars in the scene. Unity currently offers three options of scripting languages: C#, JavaScript and Boo. All game examples showed here were written in C# language. The initial learning curve of Unity for construction of scenarios and writing the scripts is quite demanding, but this pays off because of the great variety of high quality scenarios that can be produced in a short time. The Unity assets store also helps, because users can find a lot of interesting and complex materials, like characters, objects, and animations. The Unity game engine was employed here given its cross-platform availability (Microsoft Windows®, Linux and Mac OSX, web player, IOS and Android) and ease of use, but other tools, such as Unreal Development Kit (UDK, https://www.unrealengine.com/products/udk) and Cry Engine SDK (http://www.cryengine.com/), could also be potentially implemented as frontends.

#### Medieval virtual scenario frontend

**Figure 5:**
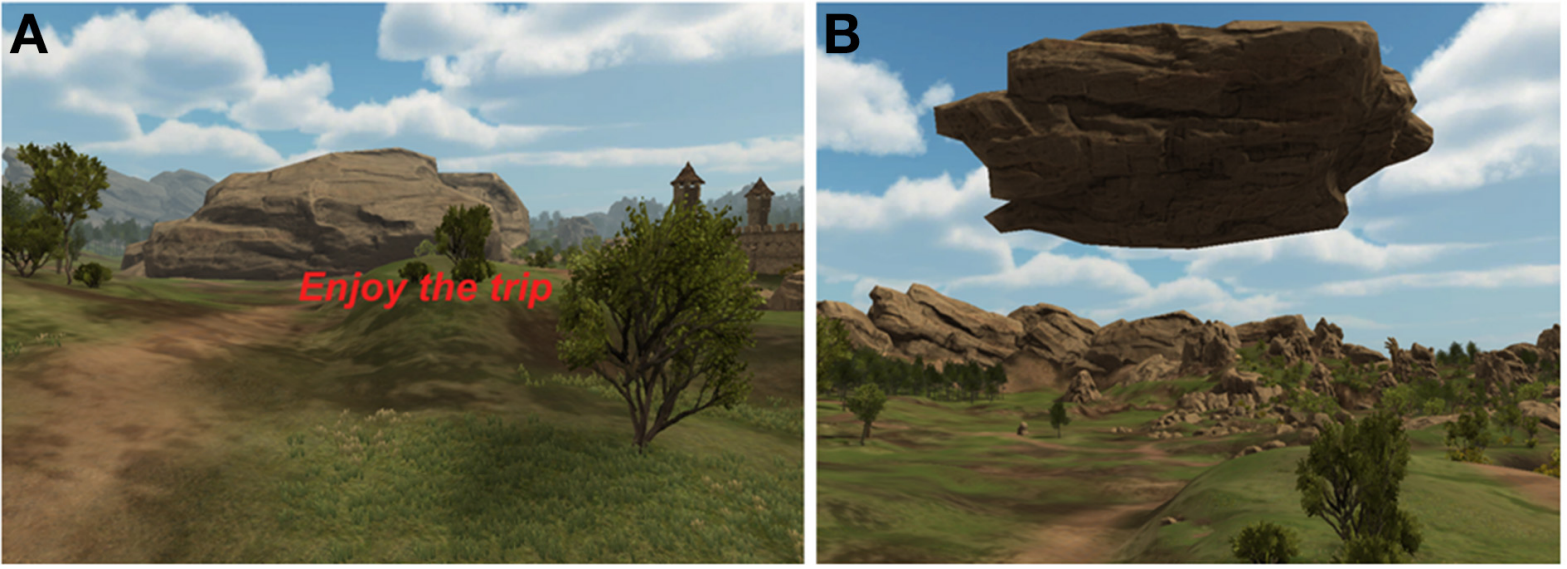
**Snapshots of the game like frontend designed in Unity, showing the path that the participant travels during the experiment. (A)** Shows the situation where the rock is blocking the way; **(B)** shows the rock being “levitated,” unblocking the way.

Figure 5 is a screenshot of a frontend made in Unity. This frontend is a medieval virtual reality scenario in which the avatar, i.e., the participant, hovers over a path and stops in predetermined locations, blocked by a massive rock. Using the same finger tapping neurofeedback procedure exemplified in the Matlab® frontend (alternating rest and finger tapping blocks), and the same libROI plug-in, the feedback information to the participant is now given in a different way. As the participant moves across the scenario and stops right before the rock, he/she needs to perform the finger tapping task as instructed (as quickly as possible). If the percentage BOLD signal change returned by the engine reaches a predefined threshold, the rock levitates, thus unblocking the path so that the journey continues. If the threshold is not reached, the player stays at the same location until the next try, i.e., the next activation block. This scenario was constructed using objects from the iTween path editor and the Big Environment pack, available in the Unity Assets store.

#### Avatar finger tapping frontend

**Figure 6:**
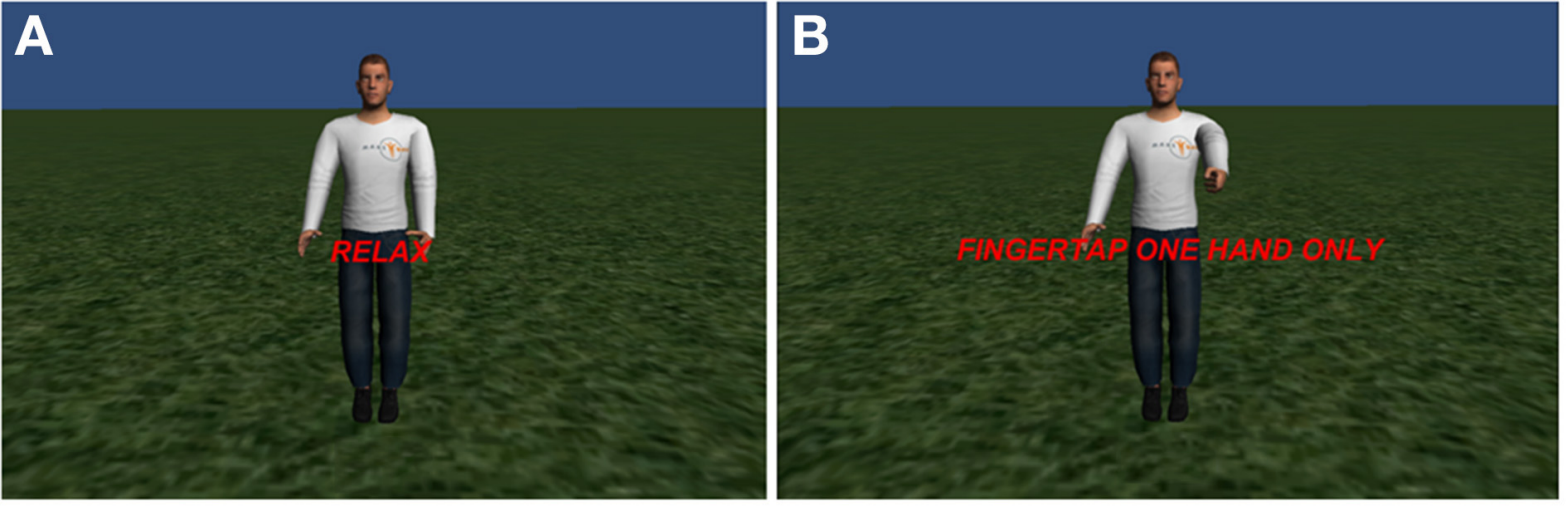
**Two situations of the avatar finger tapping scenario. (A)** During a rest block and **(B)** during a finger tapping block.

Figure 6 shows the screenshots of the avatar finger tapping frontend. It was inspired by the BOLD brain pong (Goebel et al., 2004). This is a finger tapping experiment with intercalating blocks of rest and finger tapping. Different from the previous examples, here we use two ROIs located in the primary motor cortex area of the left and right cerebral hemispheres. The participant is asked to perform finger tapping with either their left or right hand, alternating with resting blocks. This example employs the libMotor plug-in, which calculates the percent BOLD signal change in the left and right ROIs in the same way as the libROI plug-in in the single ROI example. The frontend compares the feedback values between ROIs, in such a way that the greater one will inform which hand of the avatar, showed by the frontend, will perform the finger tapping animation. If successfully performed, this conveys a clear impression that the participant is controlling the hands of the avatar with his/her own hands. The avatar and hand animations were implemented using the VR Hands Unity asset (https://serrarens.nl/passervr/downloads/vr-hands/).

## Discussion and conclusion

In this paper we introduced the FRIEND Engine framework, a reengineered implementation of our previous work (Sato et al., 2013) to provide a flexible and user-friendly framework that enables users to customize frontends and data processing pipelines for neurofeedback studies. For this aim, the standalone FRIEND software was re-implemented by breaking apart the core engine, which performs the basic data processing, the plug-in functionalities, which implement specifics of the neurofeedback study, and the frontend, which handles all the necessary graphic interfaces, external device inputs and sends commands to the engine.

The separation of the graphical interface from the data processing components provides a major advantage because users can implement virtually any type of feedback visualization strategy, such as images, audio, movie clips and virtual scenarios, by developing new frontends or connecting with standard stimulus presentation softwares and external devices. The use of the TCP/IP communication protocol between the frontend and the engine provides an interesting “weak connection,” because the frontend does not need to make any assumptions about data processing other than the ones related to the feedback interpretation. This also enables users to more efficiently share frontends that can be used for different purposes. Computer processing time is always a critical concern, especially when real-time processing and feedback are needed. All frontends herein described were run on a different computer from the one running the FRIEND Engine application. The mean processing time for one volume (single-shot EPI, 64 × 64 to 80 × 80 matrix, 22–37 slices) was about 0.7 s in mid-/high-end workstations (PC, Quad-core i7, 8 GB RAM; Macintosh, quad-core Intel Core i5, 8 GB RAM). The performance of the game frontends was not affected by the communication with the engine, and no lags were noticed for the currently implemented routines and data types.

The FRIEND Engine framework flexibility was illustrated here by the game frontends. This implementation demonstrated the potential use of virtual reality in neurofeedback studies, which may increase engagement and compliance with the tasks. The economic push of commercial games and widely available software development kits facilitates constant updates and improvements that can be quickly embedded into new frontends for enhanced neurofeedback studies. The frontend implementation examples herein provided employed the Unity game engine (https://unity3d.com/), because of its user-friendliness, flexibility and availability for the three main operational systems (Windows®, Linux and Mac OSX).

Source code is available for all the software provided within the FRIEND Engine distribution, so that seamless customization is possible. From the user point of view, there is no apparent need to modify the exhaustively tested and well-established FRIEND Engine core functionalities, though these are open for improvement. Advanced users can also modify any existing plug-in (e.g., implementing ROI correlations using more than two ROIs) or create new ones (e.g., implementing simultaneous real-time fMRI and EEG neurofeedback). A few frontends and plug-ins are provided with this first distribution, but we expect that this can be substantially expanded whenever users share their developments, with a benefit for the growing scientific community interested in neurofeedback research (Sulzer et al., 2013).

Whereas the standalone FRIEND implementation includes quality controls mechanisms (e.g., a GUI that allows users to monitor details of the ongoing acquisition, such as motion parameters, visualization of ROIs transformed from the MNI space to subject space, the temporal variation of a ROI mean), FRIEND Engine still lacks a quality control module. While some of these functionalities are currently available on the Matlab® frontend (e.g., display of motion parameters), it is not the case for the game-like frontends. Toward this aim, we are currently developing an ancillary frontend for quality control, which will run independently from the main frontend. To access the motion parameters information and possibly feedback information, this frontend will only need the ID of the session workspace created by the main frontend. This ancillary frontend module also contains visualization capabilities for displaying anatomical and functional reference images, source MNI ROIs, ROIs transformed into subject space, visualization of active voxels prior to neurofeedback based on GLM thresholds or SVM feature-selection steps, among some other options. This frontend will operate in a largely generic way, as to be capable of working in conjunction with current or future frontends. In the short term, we are also planning to deliver a Python (https://www.python.org/) and a Presentation® (http://www.neurobs.com/) frontend.

In terms of usage and availability, currently AFNI (Cox, 1996) and Turbo Brain Voyager (Goebel, 2012) appear to be the leading packages for rtMRI neurofeedback. AFNI is a highly developed fMRI package that has pioneered work on real-time neurofeedback experiments. Turbo Brain Voyager is a user-friendly fMRI processing package containing a rtfMRI module that enjoys the benefits from a number of pre- and post-processing routines and an attractive graphic interface. Similarly, our package uses fMRI spatial and temporal processing routines that are largely based on the widely used and validated FSL package. Both AFNI and Brain Voyager allow ROI processing and thermometer-like feedback, as FRIEND Engine does. In a very recent publication, Cohen et al. (2014) employed Brain Voyager and the Unity environment to enable participants to control an avatar by hand and leg motion imagery, similarly as we report here. AFNI and Brain Voyager also provide interesting quality control functionalities, which are available in the standalone FRIEND version and which are currently being implemented and expanded in FRIEND Engine. For developers, Brain Voyager allows development of plug-ins for Windows®, MAC® and Linux platforms in C++ language whereas AFNI allows the addition of run-time functionalities in C language for MAC® and Linux. For FRIEND Engine, we recommend plug-ins to be developed in C++ for compatibility. Brain Voyager is a commercial package, thus source codes are not available, whereas AFNI and FRIEND Engine Framework are open source. An optimized FSL embedded functionality allows FRIEND to run seamlessly on Windows® (the official FSL package currently does not run on this platform), so there is no need for a virtual machine. AFNI requires Cygwin (https://www.cygwin.com) to run on Windows®, although with reduced functionality and possibly reduced performance. FRIEND Engine has the added advantage of providing full platform and language freedom for the development of frontends. A recent, freely available toolbox for rt-fMRI, implements similar capabilities as those described in FRIEND Engine, Turbo Brain Voyager and AFNI, and and adds the interesting feature of “subject-independent” multivariate pattern classification (Rana et al., 2013). However, so far it has not been made widely available for download, and we have not been able to evaluate and compare it to existing ones.

With respect to future developments in FRIEND Engine, one of our main goals is to encourage clinical applications. For this aim, streamlining routines for blind randomization procedures are under way. These will allow experimenters to run double-blind randomized controlled fMRI neurofeedback trials in a rigorous and straightforward manner.

The importance of building a repository for frontends and plug-ins is clear. A publicly accessible repository, with a discussion forum for implementations and strategies can be of great help for the development of new neurofeedback projects. To this aim, sharing of plug-ins and frontends will be possible through the NITRC repository (http://www.nitrc.org/projects/friend) and GitHub (https://github.com/InstitutoDOr/FriendENGINE). In summary, we believe that FRIEND Engine can be a valuable contribution to the thriving fMRI neurofeedback community, by providing an open and flexible collaborative platform for developing new solutions for fMRI neurofeedback research and clinical applications.

### Conflict of interest statement

The authors declare that the research was conducted in the absence of any commercial or financial relationships that could be construed as a potential conflict of interest.
