# Supplementary material for: FRIEND Engine Framework: a real time neurofeedback client-server system for neuroimaging studies
Source: Front Behav Neurosci. 2015 Jan 30;9:3. doi: 10.3389/fnbeh.2015.00003 (PMC4311636; doi:10.3389/fnbeh.2015.00003)
Supplement: Supplementary file 1 [file DataSheet1.PDF]

21/Jan/2015

Supplementary Material

FRIEND Engine Framework: A real time neurofeedback client-server system for neuroimaging studies

Rodrigo Basilio[1], Griselda J. Garrido[1], João R. Sato[1,2], Sebastian Hoefle[1], Bruno R. P. Melo[1], Fabrício Pamplona[1], Roland Zahn[3], Jorge Moll[1*]

[1] Cognitive and Behavioral Neuroscience Unit and Neuroinformatics Workgroup, D'Or Institute for Research and Education (IDOR), Rio de Janeiro, Brazil
[2] Center of Mathematics, Computation and Cognition. Federal University of ABC, Santo André, Brazil
[3] Department of Psychological Medicine, Institute of Psychiatry, King's College, London, United Kingdom

*** Correspondence:** Jorge Moll (jorge.moll@idor.org). Cognitive and Behavioral Neuroscience Unit and Neuroinformatics Workgroup, D'Or Institute for Research and Education (IDOR), Diniz Cordeiro 30, Rio de Janeiro, 22281-100, Brazil.

1. Supplementary Data

To illustrate the utilization of the FRIEND Engine Framework we present code snippets from the libROI plug-in used in the Matlab frontend and in the medieval virtual scenario frontend presented in the main paper. The code snippet 1 contains the function processROI, which calls a class method that actually performs the calculations. It also demonstrates the use of some methods of the studyParameters class and volume manipulation with functions used within the FSL source code. Other functions and objects, like the one that calculates the mean of a ROI are also shown.

The targetValue variable used at the end of the method processVolume is read within the initializeROIProcessing function. The studyParams variable grants the plug-in library access to the setup configuration information of the study contained in the studyparams.txt file, read the 'READCONFIG' message command (see Table 1 in the article), including variables that are present in the configuration information but are not known by the studyParams object, allowing the plug-in library to read its own configuration variables.

```
// libROI Plug-in function that calculates the feedback value that the engine will pass to the frontend
int roiProcessing::processVolume(VariablesDatabase&vdb, int volIdx, float& classnum, float&
feedback)
{
  char processedFile[200];
  int idxInterval=vdb.interval.returnInterval(volIdx);
```

```
  volume <float> vol;
  // gets the motion corrected and Gaussian smoothed file
  vdb.getMCGVolumeName(processedFile, volIdx);
  read_volume(vol, string(processedFile));

  classnum = vdb.getClass(volIdx);
  feedback = 0;

  // if in baseline condition, adds current image to the previous sum image
  if (vdb.interval.isBaselineCondition(volIdx))
  {
    if (vdb.interval.intervals[idxInterval].start == volIdx)
      baselineMean= vol;
    else
      baselineMean+= vol;
    // when finishing adding images on baseline condition block,
    // divide the sum image by the size of the block to create the mean volume
    if (vdb.interval.intervals[idxInterval].end == volIdx)
    {
      baselineMean /= (vdb.interval.intervals[idxInterval].end-
          vdb.interval.intervals[idxInterval].start+1);
      // obtain ROI mean value MeanCalculation object variable was initialized
      // previously with the ROI mask in Initialization function.
      // calculateMeans obtains the mean for each ROI in the current volume
      meanCalculation.calculateMeans(baselineMean);

      // Calculates the mean of the ROI for the current mean volume.
      lastBaselineValue=meanCalculation.means[0];
    }
  }
  else
  // task condition. Taking the mean of the volume ROI and calculating the PSC
  {
    meanCalculation.calculateMeans(vol);
    // Percent signal change calculation
    feedback=PSC(meanCalculation.means[0], lastBaselineValue);
    // Divide feedback value by user-defined target value. The front-end
    // will use the feedback value for neurofeedback display
    feedback= feedback /targetValue;
  }
  return 0;
}

// plug-in function for the calculation of the feedback value
extern "C" int processROI(VariablesDatabase& vdb, int index, float&classnum, float& projection,
void*& userData)
{
```

```
  roiProcessing *roiVar =(roiProcessing *) userData;
  roiVar->processVolume(vdb, volIdx, classnum, projection);
  return 0;
}
```

Code snippet 1. Main function of the libROI plug-in that calculates the feedback value.

The following code snippets are from the Matlab® frontend showing how information must be exchanged with the engine via a non-blocked TCP/IP communication in the ROI processing pipeline. The first command issued is the "NEWSESSION" command that creates a new session in memory and returns the session id that uniquely identifies the newly created session. As already explained in the main paper, a session is an independent location in the memory of the computer running the engine, capable of storing all the information needed to be sent back to the frontend, such as neurofeedback information and motion corrected volume parameters. We use the Matlab functions fprintf and fgetl to send and receive, respectively, messages via TCP/IP communication.

The variables mainThread and responseThread, presented in the following code snippets, are two Matlab® TCP/IP objects. The connection established through the mainThread variable is the first made and lasts until the acquisition run processing is ended. The principal commands are sent through that connection. The connection established through the responseThread is temporary. Each time an information is needed, such as the feedback information for a specific volume, the connection is opened and after the information is acquired, the connection is closed.

```
fprintf(mainThread,'NEWSESSION');
% reading session id
sessionID=fgetl(mainThread);
% reading acknowledge
response=fgetl(mainThread);
```

Code snippet 2. Creating a new session in the engine

The next command issued by the frontend is the "PLUGIN", which sends the plug-in library filename and the names of the functions that the engine must call to accomplish the objective of the neurofeedback study. This list of function names must be sent in a pre-specified order (train, test, initialization, finalization, volume and post-processing as indicated by the Matlab® % commentary directives to the right of each programming line). Not implemented functions must be specified as "no" in the place where the name of the function should be present.

```
% sending the PLUG-IN command and parameters
fprintf(mainThread,'PLUGIN');
fprintf(mainThread,'libROI');
fprintf(mainThread,'no');% train function
```

```
fprintf(mainThread,'processROI');% test function
fprintf(mainThread,'initializeROIProcessing');% initialization function
fprintf(mainThread,'finalizeProcessing');%finalization function
fprintf(mainThread,'no');% volume function
fprintf(mainThread,'no');% post preprocessing function
% getting the acknowledge
response=fgetl(mainThread);
```

Code snippet 3. Configuring the plug-in to be used in the experiment

The next command is “NBPREPROC”, which initiates the preprocessing steps of the FRIEND pipeline in asynchronous (non-blocked) mode. Note that after this command, the frontend has to regularly query the engine for the termination of this step.

```
% sending PREPROC non-blocked command
fprintf(mainThread,'NBPREPROC');
% getting the acknowledge
response=fgetl(mainThread);
```

Code snippet 4. Starting the PREPROC asynchronously

```
% open a communication channel with the engine
fopen(responseThread);
% sending the session command to create a new workspace
fprintf(responseThread,'SESSION');
% sending the session id
fprintf(responseThread,'%s',sessionID);
response=fgetl(responseThread);
% sending the TEST sub-command
fprintf(responseThread,'TEST');
% sending the volume index of the feedback value
fprintf(responseThread,'%d',actualVolume);
% getting feedback information
class=str2double(fgetl(responseThread));
percentage=str2double(fgetl(responseThread));
% getting acknowledge
response=fgetl(responseThread);
% closing the connection
fclose(responseThread);
```

Code snippet 5. Getting a feedback value

Next, the “NBFEEDBACK” command is sent, initiating the processing of the volumes of the actual run. The frontend client issues various SESSION/GRAPHPARS commands through the responseThread variable to query for translation and rotation parameters of each motion corrected

volume to be presented in graphs placed in the interface, and SESSION/TEST commands to control the thermometer to be presented to the participant, as showed in code snippet 5. If all the volume files in the run were processed, the SESSION/GRAPHPARS command returns an "END" token, indicating the termination of the feedback process.
